# Optimal spectrum granularity for spectrally-efficient elastic optical networks

**DOI:** 10.1038/s41598-026-49539-3

**Published:** 2026-04-22

**Authors:** Ujjwal Ujjwal, Shrinivas Petale, Jaisingh Thangaraj

**Affiliations:** 1 Manipal Institute of Technology, Manipal Academy of Higher Education, Manipal, India; 2 Department of Electrical and Computer Engineering, The George Washington University, Washington, DC USA; 3 Department of Electronics Engineering, Indian Institute of Technology (Indian School of Mines), Dhanbad, Jharkhand 826004 India

**Keywords:** Elastic optical network, Routing and spectrum assignment, Flexible grid, MILP, Slot width, Network cost, Engineering, Mathematics and computing, Physics

## Abstract

In this paper, we compute the optimal slot width which provides a good trade-off between the blocking probability and network cost, aiming to achieve a spectrally and cost-efficient solution for Elastic Optical Networks (EONs). Here we have determined the blocking performance by varying the slot width from 50 GHz down to 1.5625 GHz, followed by network cost estimation considering various network elements, namely transponders and node equipment. The analysis reveals that reducing the slot width from 50 GHz to 6.25 GHz leads to a substantial reduction in blocking probability, approximately 70%, with an estimated normalized cost increment of approximately 0.6. Further reductions to 3.125 GHz and 1.5625 GHz offer only minor additional improvements in blocking probability, while incurring normalized cost increments that are disproportionate to the minor additional performance gains, rising to approximately 0.75 and 1.0 (relative to the 50 GHz baseline). These results indicate that narrowing the slot width beyond 6.25 GHz leads to diminishing returns in performance at a substantially higher cost. Based on this trade-off analysis, 6.25 GHz is identified as the optimal slot width, providing the best balance between blocking performance improvement and network cost efficiency.

## Introduction

The demand for bandwidth has been increasing rapidly, driven by emerging services like video conferencing, social networking, multimedia, and cloud computing. This surge in traffic continues to escalate daily with ongoing technological advancements. As a result, network operators need to implement next-generation optical networks that can handle the growing traffic demand while ensuring cost efficiency and operational flexibility^1,2^. To address these high traffic demands and the need for fine spectrum granularity, Elastic Optical Networks (EONs) have been introduced to provide dynamic bandwidth allocation capabilities^3^. By utilizing flexible grid technology, EONs address the constraints of traditional fixed-grid Dense Wavelength Division Multiplexing (DWDM) systems and significantly enhance the efficiency of connection provisioning. In this context, the term elastic primarily refers to two key characteristics: (i) flexible spectrum allocation, and (ii) the ability to establish optical paths that support variable data rates.

Recent studies have identified EONs as a promising approach for efficiently managing spectral resources, leading to more effective spectrum utilization. In flexible-grid EONs, the fixed 50 GHz channel defined by the International Telecommunication Union (ITU) is subdivided into narrower frequency slots (FSs), ranging from 50 GHz down to 1.5625 GHz. In EONs, contiguous FSs are assigned to connection requests based on their bandwidth requirements and the selected modulation format^4,5^. This flexibility makes EONs particularly effective in supporting bandwidth-intensive and diverse applications, including big data analytics, cloud-based services, and high-performance distributed computing^6,7^. The elastic characteristics of the EON can vary the slot width from a few GHz to narrower, and recent advancements in filtering technologies have allowed shifting even to finer spectrum granularities. A large slot width can result in spectrum wastage and reduced network capacity, particularly for lower data rate connections, whereas a smaller slot width improves blocking performance and enables more efficient utilization of spectral resources^8,9^. On the contrary, smaller slot width increases the hardware complexity of reconfigurable optical add-drop multiplexer (ROADM) equipment viz; tunable laser, tunable filter, spectrum selective switch (SSS), power amplifiers and as well as increase the complexity of the software-controlled network management system^10^. While prior works have explored spectrum granularity and its impact on blocking probability and hardware complexity, they have primarily focused on fine granularities and have not examined ultra-fine slot widths in detail. Additionally, they have not provided a quantitative evaluation of the cost impact associated with using narrower slot widths.

This paper addresses the current gap by conducting a comprehensive analysis of both blocking performance and network cost as slot widths are reduced from 50 GHz down to 1.5625 GHz, entering the ultra-fine spectrum granularity range in EONs. The study evaluates how blocking probability and cost behave under varying traffic loads and explicitly analyzes both low and high data rate connections to assess performance across diverse network scenarios. This dual-focus approach reveals the trade-offs between improved spectral efficiency and the increased hardware and deployment costs associated with narrow slot widths.

Building on this, the study offers practical guidance for real-world deployment decisions in EONs. Rather than defaulting to extremely narrow slot widths, which increase system cost without proportionate gains in performance, the findings support the adoption of more balanced and cost-effective slot width configurations, particularly in data-intensive environments such as cloud infrastructure and scientific research networks.

In this paper, we have investigated the optimal slot width in EONs for efficient utilization of spectral resources with the least hardware complexity. Initially, spectral efficiency and hardware complexity are analyzed for finer spectrum granularity. Thereafter, blocking performance is evaluated by varying the slot width from 50 GHz down to 1.5625 GHz, followed by network cost estimation considering various network elements, namely transponders and node equipment. To ensure a comprehensive evaluation, both low and high data rate connections are examined. For high data rate scenarios, the C+L band is considered to accommodate the additional spectrum requirements. The results highlight the optimal slot width that provides a balance between blocking performance and network cost.

The rest of the paper is structured as follows: “Related works” provides a review of related works, followed by the analysis of the effect of a change in the spectral width of the frequency slot in “Analysis of the effect of change in spectral width of frequency slot”. “Blocking probability and network cost estimation” introduces blocking probability and network cost estimation. “Performance analysis” presents the performance analysis, and Section 6 concludes the paper.

## Related works

Research communities are increasingly focusing on the impact of adopting finer spectrum granularity on network performance. In Ref.^11^, the authors investigated the impact of slot width and spectrum fragmentation in flexible-grid optical networks. They simulated various slot widths (ranging from 50 to 6.25 GHz) and found that a 10 GHz slot width offered the most efficient spectrum utilization, as it aligns with the greatest common factor (GCF) of typical signal bandwidths. They also showed that suboptimal slot-width choices can lead to increased fragmentation and higher blocking probabilities. However, their analysis was limited to a small set of fixed slot widths and demand types, without examining broader traffic patterns or dynamic network conditions. In our work, we extend their study by evaluating blocking performance across a wider range of slot widths and demand distributions, offering a more comprehensive understanding of how slot-size selection influences spectrum efficiency and network performance.

In Ref.^12^, the authors investigated the potential of gridless wavelength assignment to reduce optical spectrum contention in Coherent Optical Orthogonal Frequency Division Multiplexing (CO-OFDM) optical networks. They proposed a wavelength and spectrum assignment algorithm applicable to both gridless and mini-grid scenarios. Simulation results demonstrated that gridless and mini-grid-based approaches provide significantly better performance compared to conventional ITU-T grid systems. Furthermore, they showed that mini-grid systems with fine-tuning resolution can achieve performance close to that of gridless systems. However, they did not examine the blocking performance across different slot widths or quantify how spectral granularity impacts overall network performance. In our work, we analyze the blocking performance at various slot widths, providing a more detailed and quantitative understanding of the trade-offs involved in slot-size selection. These insights are essential for practical network design and optimization. In Ref.^13^, the authors evaluated the impact of slot width on flexible optical networks under various bandwidth distributions. They found that a 1 GHz slot size offers the lowest blocking probability and best spectrum utilization, but increases node complexity. Slot widths of 6.25 GHz and 12.5 GHz were identified as near optimal, offering a good balance between performance and overhead. In contrast, our work extends the performance evaluation by varying the slot width from 50 GHz down to 1.5625 GHz, and further incorporates a network cost estimation to identify the optimal slot width that balances both performance and cost. In Ref.^14^, authors conducted a comprehensive network planning study comparing three bandwidth granularity scenarios: coarse (37.5/75/112.5/150 GHz), medium (12.5 GHz steps), and fine (3.125 GHz steps). Their findings revealed that while finer granularity offers some benefits, the savings in terms of required lightpaths were only marginal (up to 5%) compared to the significant gains achieved through modulation rate adaptivity. This suggests that beyond a certain point, further refinement of spectrum granularity yields diminishing returns. In Ref.^15^, the authors investigated flexible optical networking and found that spectrum granularities of 2.5 to 3.125 GHz offer good performance, with minimal additional gains in a fully gridless scenario. In contrast, our work analyzes finer granularity down to 1.5625 GHz under both low and high data rates. We also evaluate performance under dynamic traffic and multi-band (C+L) configurations to assess spectrum utilization and blocking behavior in practical settings.

In Ref.^16^, authors investigated the impact of filter characteristics and WDM grid granularity on network-level performance in flexible Nyquist-WDM systems. Their findings indicate that a frequency granularity of 6.25 GHz provides an optimal balance between network efficiency and the complexity of filter design when allocating spectrum to both single-carrier and super-channel transmissions. In contrast, sub-channel allocation within super-channels benefits from finer granularity of 3.125 GHz, which aligns with the resolution capabilities of filters in the 1–1.2 GHz range. Improvements beyond this level of resolution and granularity show minimal additional performance benefits.

In Ref.^17^, the authors addressed the limitations of fixed grid networks in handling growing core network traffic beyond 100 Gbps. They proposed using Superchannels in Flexgrid networks to improve capacity. By comparing 50 GHz and 12.5 GHz frequency granularities, they showed that finer granularity can increase network capacity by up to 105% at 100 Gbps, though this advantage reduces to 20% at 1000 Gbps with a 0 dB link margin.

In Ref.^18^, the authors investigated the impact of spectrum granularity on the cost and efficiency of OFDM-based EONs compared to fixed-grid networks. They provided direct cost comparisons, showing that the finer granularity of EONs enables more efficient bandwidth use and potential equipment cost savings. The study demonstrated that EONs offer a cost-effective solution for increasing traffic demands while improving spectral efficiency. However, their cost computation did not consider the effects of very fine spectrum granularity. In Ref.^19^, the authors developed a multilayer IP/MPLS-over-flexgrid network design using an Integer Linear Programming (ILP) formulation combined with a Greedy Randomized Adaptive Search Procedure (GRASP) metaheuristic. Their study evaluated the cost impact of different FS widths through simulations with realistic network scenarios. They found that finer slot widths, like 12.5 GHz, are cost-effective for traffic with many low bit-rate demands but less so for long-term high bit-rate traffic. Both 12.5 GHz and 25 GHz slot widths were identified as suitable for future flexgrid networks. In Ref.^20^, the authors proposed a cost-effective transition optical cross-connect (TOXC) architecture that reuses existing WSSs in a two-stage design to reduce reliance on expensive SSSs in EONs. With the proposed contention-aware spectrum allocation (CASA) scheme, they achieved around 50% reduction in capital cost during the WDM-to-EON transition, with minimal impact on blocking performance. These findings emphasize the critical cost challenges in transitioning from WDM to finer-granularity EONs, particularly when operating at spectrum granularities of 12.5 GHz or lower. In Ref.^21^, the authors analyzed the impact of frequency grid selection on the capital expenditures (CAPEX) of multilayer IP/MPLS-over-EONs. Their results demonstrated that narrower frequency grids (e.g., 12.5 GHz and 6.25 GHz) allow for more efficient optical-layer grooming, which can significantly reduce the reliance on costly IP/MPLS equipment. However, this benefit comes at the expense of requiring more advanced and expensive bandwidth-variable wavelength selective switches (BV-WSSs). Using simulations on the Spanish Telefónica network, they found that while finer grids offer significant cost savings in low-traffic scenarios, this advantage decreases as traffic demands grow. The study suggests that moderate grids, such as 12.5 GHz or 25 GHz, provide a better long-term cost-performance balance.

In Ref.^22^, authors discuss the impact of slot width in flex-grid elastic optical networks. The authors show that adopting a 37.5 GHz slot (three 12.5 GHz slots) can increase aggregated throughput by up to 33% compared to 50 GHz fixed-grid channels. They also highlight performance trade-offs of narrower slots, including filter cascade effects causing spectral distortions and OSNR penalties. In Ref.^23^, the authors investigated an EON employing coarse-granular routing based on a newly developed optical cross-connect (OXC) architecture using coarser-granular spectrum selective switches. The study examined the impact of spectrum granularity on both network performance and cost, and compared the proposed approach with traditional WDM networks as well as conventional elastic optical networks. The results demonstrated that coarse-granular routing can achieve significant spectrum savings while reducing hardware complexity, highlighting the trade-offs between granularity, cost, and spectrum utilization.

For clarity, in EONs, key switching components include OXCs, Wavelength Selective Switches (WSSs), SSSs, and BV-WSSs, which operate at different switching granularities. OXCs enable end-to-end bandwidth-variable lightpath provisioning by routing and grooming spectral resources across the network. WSSs route individual wavelength channels and serve as the core switching fabric in conventional WDM-based ROADM/OXC nodes. SSSs support finer spectrum-level switching at sub-wavelength granularity, offering greater spectral flexibility than conventional WSSs for efficient spectrum management in EONs. BV-WSSs further extend this capability by enabling allocation of variable-sized frequency slots for elastic transmission, dynamically adjusting the switched bandwidth to match variable-rate transponders.

## Analysis of the effect of change in spectral width of frequency slot

In this section, we analyze how changes in slot width affect several key network parameters. A major challenge in flexible-grid optical network design is selecting an appropriate spectrum granularity (slot width). Employing finer spectrum granularities, such as 6.25 GHz, 3.125 GHz, and 1.5625 GHz, can reduce spectrum wastage and improve spectrum utilization, but also increases network complexity and hardware costs. Therefore, it is essential to identify an appropriate trade-off among slot width, blocking performance, and overall network cost. The impacts on spectral efficiency and hardware complexity are further explored in the following subsections.

### Effect on spectral efficiency

ROADMs require tunable optical filters on the drop side to support variable bit rate signals. These filters must be precisely tuned in both center frequency and passband bandwidth to accommodate flexible spectrum allocations. As spectrum granularity becomes finer, the required filter bandwidth decreases, making it more challenging to maintain sharp roll-off characteristics. This can lead to increased spectral overlap and potential inter-channel interference^24^.

In addition to optical filtering in ROADMs, pulse shaping at the transmitter and receiver plays a critical role in minimizing inter-symbol interference and optimizing spectral efficiency. Root Raised Cosine (RRC) pulse shaping filters are widely used in the digital signal processing (DSP) modules of coherent optical systems. The RRC filter satisfies Nyquist’s first criterion, enabling zero inter-symbol interference under ideal conditions. Its frequency response can be expressed as shown in Eq. (1)^25,26^.

1 $$\begin{aligned} S(f) = {\left\{ \begin{array}{ll} T, & 0 \le |f |\le \frac{1 - \mu _i}{2T} \\ \frac{T}{2} \left[ 1 + \cos \left( \frac{\pi T}{\mu _i} \left( |f |- \frac{1 - \mu _i}{2T} \right) \right) \right] , & \frac{1 - \mu _i}{2T} < |f |\le \frac{1 + \mu _i}{2T} \\ 0, & |f |> \frac{1 + \mu _i}{2T} \end{array}\right. } \end{aligned}$$

where *T* represents the Symbol period and $\mu _i$ represents the roll–off factor.

The roll-off factor helps determine the filter’s bandwidth and the sharpness of its transition band. Reducing the roll-off factor results in tighter bandwidth (band-limiting) but leads to steeper filter transitions, which can cause increased signal distortion and sensitivity to timing errors. This, in turn, impacts the spectral efficiency and the required guard band in the system^27^.

**Fig. 1:**
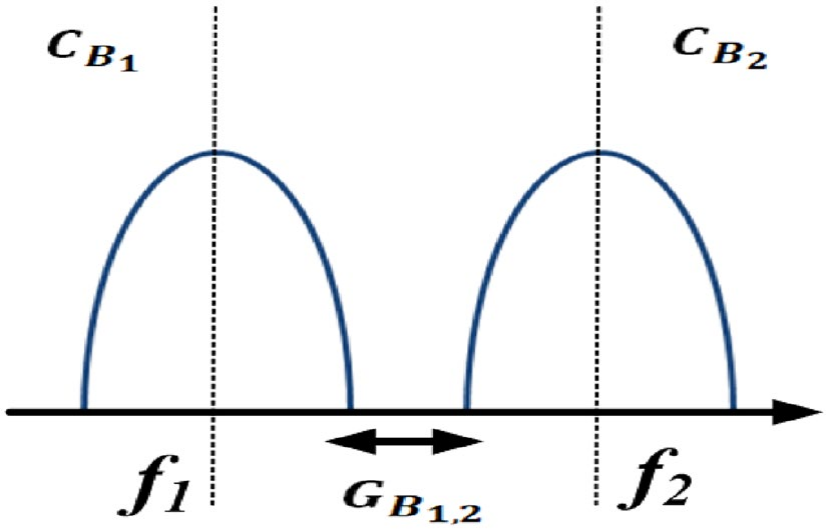
Illustrates the relationship between channel bandwidth and guard band in a flexible grid optical network.

2 $$\begin{aligned} \Delta f = (f_{2} - f_{1}) \end{aligned}$$

Thus, guard-band is given by the Eq. (3).

3 $$\begin{aligned} G_{B_{1,2}} = \Delta f - \frac{1}{2} (C_{B_1} + C_{B_2}) \end{aligned}$$

where $G_{B_{1,2}}$ is the guard-band between channel 1 and 2, $C_{B_1}$ represents occupied channel bandwidth of the first channel, centered at frequency $f_1$, and $C_{B_2}$ represents the occupied channel bandwidth of the second channel, centered at frequency $f_2$. In this study, the occupied channel bandwidths $C_{B_i}$ are determined by the null-to-null bandwidths of the RRC pulse-shaping filters, as given by Eq. (4).

4 $$\begin{aligned} C_{B,i} = \frac{1 + \mu _i}{T} \end{aligned}$$

$\Delta f$, as defined in Eq. (2), represents the frequency separation between two carrier frequencies (as illustrated in Fig. 1). In this study, the channel bandwidths $C_{B_1}$ and $C_{B_2}$ are determined based on the bandwidth of the RRC pulse-shaping filter.

By substituting Eq. (4) into Eq. (3), the guard-band can be expressed as a function of the roll-off factor. (It is assumed that the roll-off factors are equal for all channels.) The resulting expression is provided in Eq. (5).

5 $$\begin{aligned} G_{B_{1,2}} = \Delta f - \frac{1}{2} \left( \frac{1 + \mu _1}{T} + \frac{1 + \mu _2}{T} \right) \end{aligned}$$

6 $$\begin{aligned} G_{B_{1,2}} = \Delta f - \frac{1}{2T} \left( 2 + \mu _1 + \mu _2 \right) \end{aligned}$$

**Fig. 2:**
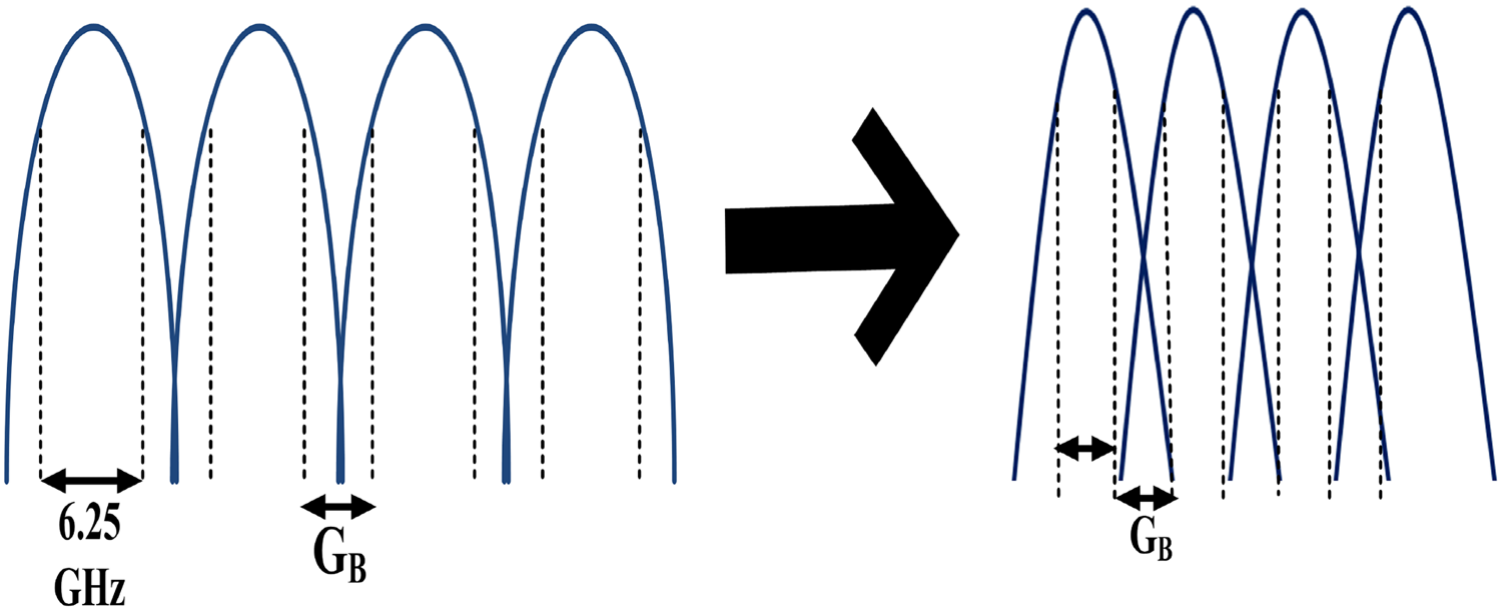
Effect of decreasing the slot width on guard-band.

Based on Eq. (6), the guard-band requirement is inversely proportional to the roll-off factor. Finer spectrum granularity requires narrow-band filters, which are typically designed with low roll-off factors to achieve sharp spectral transitions. However, low roll-off factors increase the guard-band needed to mitigate adjacent channel interference (ACI). Consequently, a significant portion of the spectrum may be occupied by guard bands, potentially diminishing the spectral efficiency advantages expected from finer spectrum granularity, ultimately making the system spectrally inefficient as shown in Fig. 2.

### Effect on hardware complexity

A larger slot width leads to the wastage of spectral resources and limits network capacity, while smaller slot widths improve spectral resource utilization. But hardware facilities such as fine grid (3.125 GHz, 1.5625 GHz) SSSs, tunable lasers, tunable filters, and high power amplifiers are challenging and costly to manufacture.

WSS are essential components of OXCs. In EONs, the traditional wavelength-selective switch (WSS) used in fixed grid systems is replaced by SSS. The SSS operates similarly to the WSS but offers finer spectral granularity, enabling more flexible and efficient spectrum allocation. Assume that the granularity factor is taken as *g* and the device supports *W* channels. Channels commonly conform to the 50 GHz or 100 GHz ITU grid across the device’s spectral range. The granularity factor *g* defines how many smaller spectral slots (e.g., 12.5 GHz or 6.25 GHz) are contained within each channel. Thus, an SSS can independently configure *gW* FSs, providing fine-grained spectral control. Hence, the flexibility of the $1\times K$ SSS can be expressed as^28^.

7 $$\begin{aligned} F(S) = gW \, \, {log(K+1)} \end{aligned}$$

Here, *F*(*S*) represents the static switching flexibility of an SSS, quantifying how many distinct configurations it can support at a given time. It reflects the SSS’s ability to independently route *gW* fine-grained spectral slots to different output ports. Each spectral slot can be configured in K + 1 distinct ways: it may be routed to any one of the K output ports, or left unrouted (i.e., blocked). This accounts for all possible per-slot switching actions, which together determine the overall configurability of the device. A higher F(S) means greater adaptability to diverse traffic demands, enabling efficient spectrum use and fine-tuned switching in elastic optical networks.

Switching flexibility is defined as the ability of a device to dynamically reconfigure and route FSs between different output ports over time. If the SSS is reconfigured *z* times over a period *T*, referred to as the transition rate $\left( \frac{z}{T}\right)$, then the switching flexibility of the SSS over time *T* is given by

8 $$\begin{aligned} F(S,T) = z \cdot gW \cdot \log (K+1) \end{aligned}$$

The expression *F*(*S*, *T*) extends the concept of flexibility to the temporal domain by considering how often the SSS can reconfigure within a time period T, where *z* is the number of reconfigurations. In practical terms, this reflects the device’s ability not only to support a wide range of spectral configurations, but also to dynamically adapt them over time in response to changing traffic patterns, failures, or network optimization needs. A higher F(S,T) implies both fine-grained spectral control and high responsiveness, enabling the SSS to play a key role in real-time traffic engineering and dynamic resource allocation in flexible optical networks.

Let $B_{s}$ is the total spectral bandwidth then the slot width ($f_{slot}$) can be expressed as $\frac{B_{s}}{\left( gW \right) }$. Using this relation Eq. (8) can be represented in terms of slot width^29^.

9 $$\begin{aligned} F(S,T) = z \cdot \Big (\frac{B_{s}}{f_{slot}} \Big ) log(K+1) \end{aligned}$$

Equation (9) shows that the switching flexibility of SSS is inversely proportional to the spectrum width of the slot. Thus, higher switching flexibility is required for supporting finer spectrum granularity (e.g., 1.5625 GHz). But achieving high switching flexibility is very strenuous and expensive. This is because a reduction in slot width increases the transition rate ($\left( \frac{z}{T}\right)$), meaning the SSS must reconfigure more frequently to accommodate a larger number of spectral slots within a fixed bandwidth.

However, achieving such high reconfiguration rates is challenging in practice due to hardware limitations, such as finite switching speed, control latency, and stability of the switching elements. As the transition rate increases, maintaining reliable and precise switching becomes harder. In addition, ultra-fine spectrum granularity imposes strict requirements on the spectral resolution of SSSs, which must provide sharp filtering and high selectivity. This leads to practical issues such as increased insertion loss, filter imperfections, and inter-channel crosstalk.

Consequently, the combination of fine spectral granularity and high transition rates significantly increases system complexity, power consumption, and implementation cost. While finer spectrum granularity improves theoretical flexibility and spectral efficiency, it introduces substantial technological challenges that limit its practical feasibility with current hardware.

Furthermore, both tunable lasers and tunable optical filters must be precisely adjusted to support finer spectrum granularity. For example, even at a slot width of 12.5 GHz, tunable lasers are typically required to provide a fine-tuning resolution (e.g., 6.25 GHz) to accurately align each signal with the center frequency of its allocated spectral slot. As the slot width decreases further (e.g., to 6.25 GHz or below), the requirements on frequency resolution, stability, and tuning accuracy become significantly more strict.

This increased precision makes the system more sensitive to frequency drift, phase noise, and filter imperfections. As a result, improved control mechanisms and high-resolution components are necessary to maintain proper spectral alignment and prevent overlap between adjacent channels. Moreover, tunable optical filters must have sharper roll-off characteristics and higher selectivity to effectively confine signals within narrower bandwidths. These strict requirements lead to more complex hardware, higher power consumption, and high implementation costs. Therefore, while finer spectral granularity improves spectral efficiency, it also introduces significant challenges in terms of practical hardware realization.

Thus, an optimal slot width that maintains a good trade-off between blocking probability and network cost needs to be identified. In the following section, we performed a blocking probability and network cost-based analysis to determine an optimal slot width^30^.

## Blocking probability and network cost estimation

**Fig. 3:**
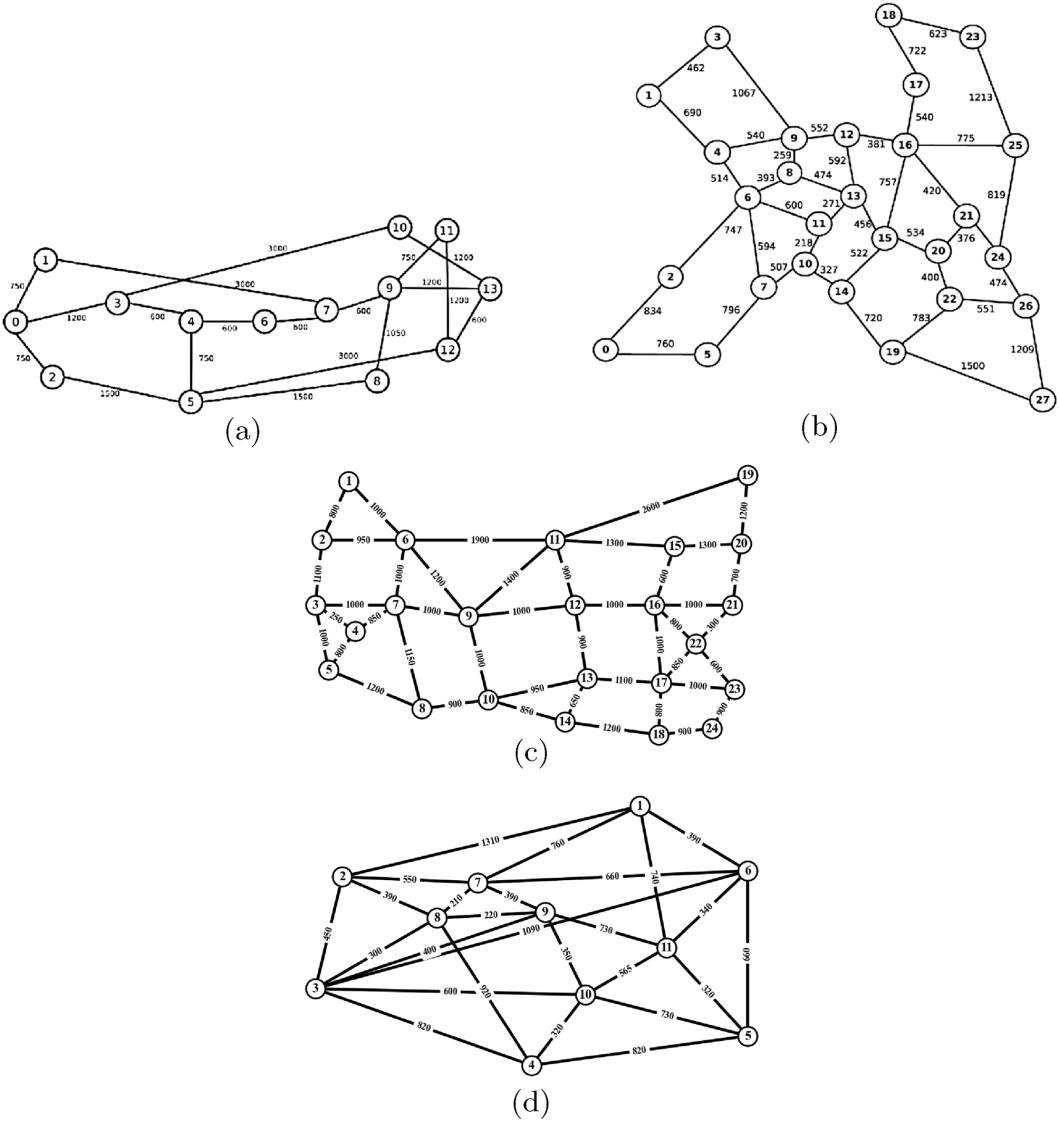
(**a**) 14-node, 21-link NSFNET topology; (**b**) 28-node, 41-link PAN-EUROPEAN topology; (**c**) 24-node, 43-link USNET topology; and (**d**) 11-node, 26-link COST 239 topology.

In this section, we investigate the optimal slot width by jointly evaluating blocking probability and network cost at different slot widths over the considered network topologies (as shown in Fig. 3).

### Blocking probability

In this section, we present Algorithm 1, which performs dynamic routing and spectrum assignment (RSA) in EONs to evaluate the blocking performance under different slot widths. Here, the slot width is varied from 50 GHz to 1.5625 GHz to evaluate the network’s performance in terms of blocking probability.

The EON topology can be expressed as a graph *G*(*V*, *E*), where *V* represents the set of nodes and *E* represents the set of fiber links. It is assumed that all FSs have equal bandwidth, and each fiber link accommodates *F* FSs. The capacity of an FS is given by $M \times f_{slot}$ Gb/s, where *M* is the modulation level and $f_{slot}$ is the bandwidth of the FS. The modulation level *M* can take values of 1, 2, 3, or 4, corresponding to BPSK, QPSK, 8-QAM, and 16-QAM, respectively. A dynamic request is represented as $C_r$(*s*, *d*, $Q_{c}$), where *s* and *d* correspond to the source and destination nodes, respectively, and $Q_{c}$ Gb/s indicates the required bandwidth for the connection. It is assumed that spectrum conversion is not permitted within the network; therefore, *N* contiguous FS must occupy the same spectrum on each link, in accordance with the spectrum continuity constraint.

**Fig. 4:**
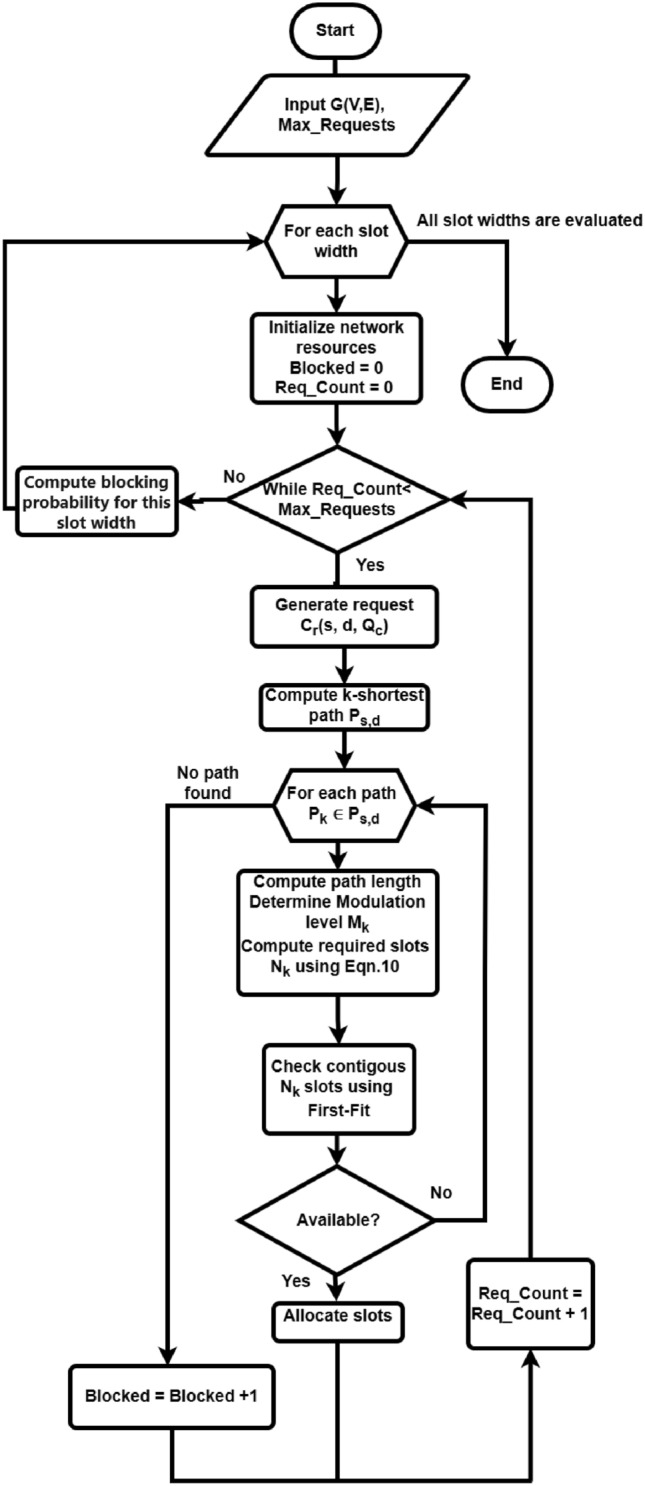
Flowchart illustrating the workflow of the proposed algorithm.

The required number of contiguous FSs to establish a connection request can differ depending on the selected routing path. Hence, it is determined by using Eq. (10).

10 $$\begin{aligned} N = \Bigg [\frac{Q_{c}}{M \times f_{slot}}\Bigg ] + N_{g} \end{aligned}$$

where *N* represents the number of contiguous FS, $N_g$ denotes the number of FSs allocated as a guard band to prevent interference between adjacent spectrum allocations. In this work, we set $N_g$=1, following common practice in elastic optical network design, where a single FS is typically reserved as a guard band. This choice ensures sufficient spectral isolation between adjacent spectrum allocations and aligns with typical system-level requirements. Here the value of $f_{slot}$ is varied from 50 GHz down to 1.5625 GHz^31^ (Fig. 4).

**Algorithm 1:**
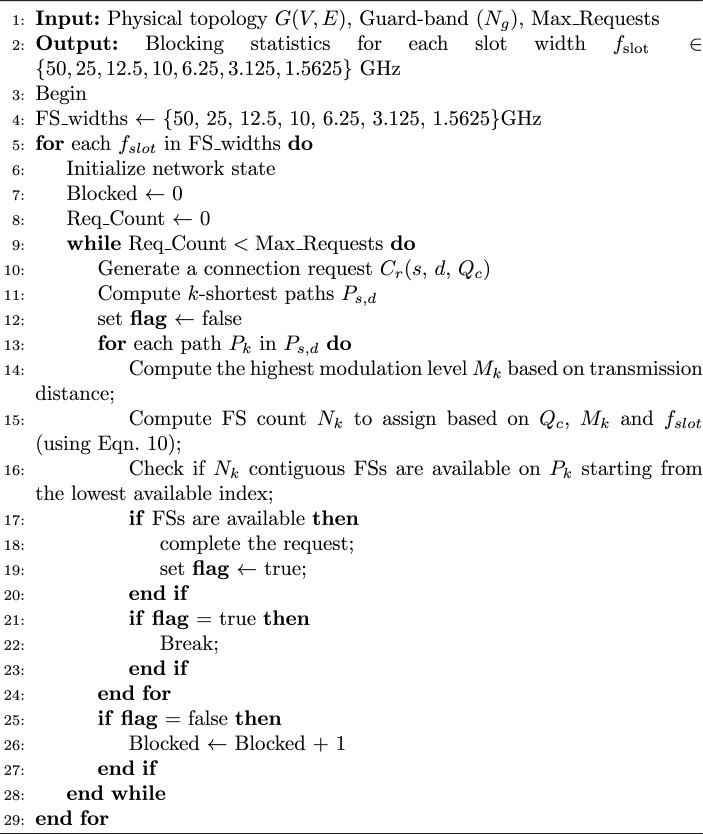
Routing and spectrum assignment algorithm (RSA)

### Network cost

**Fig. 5:**
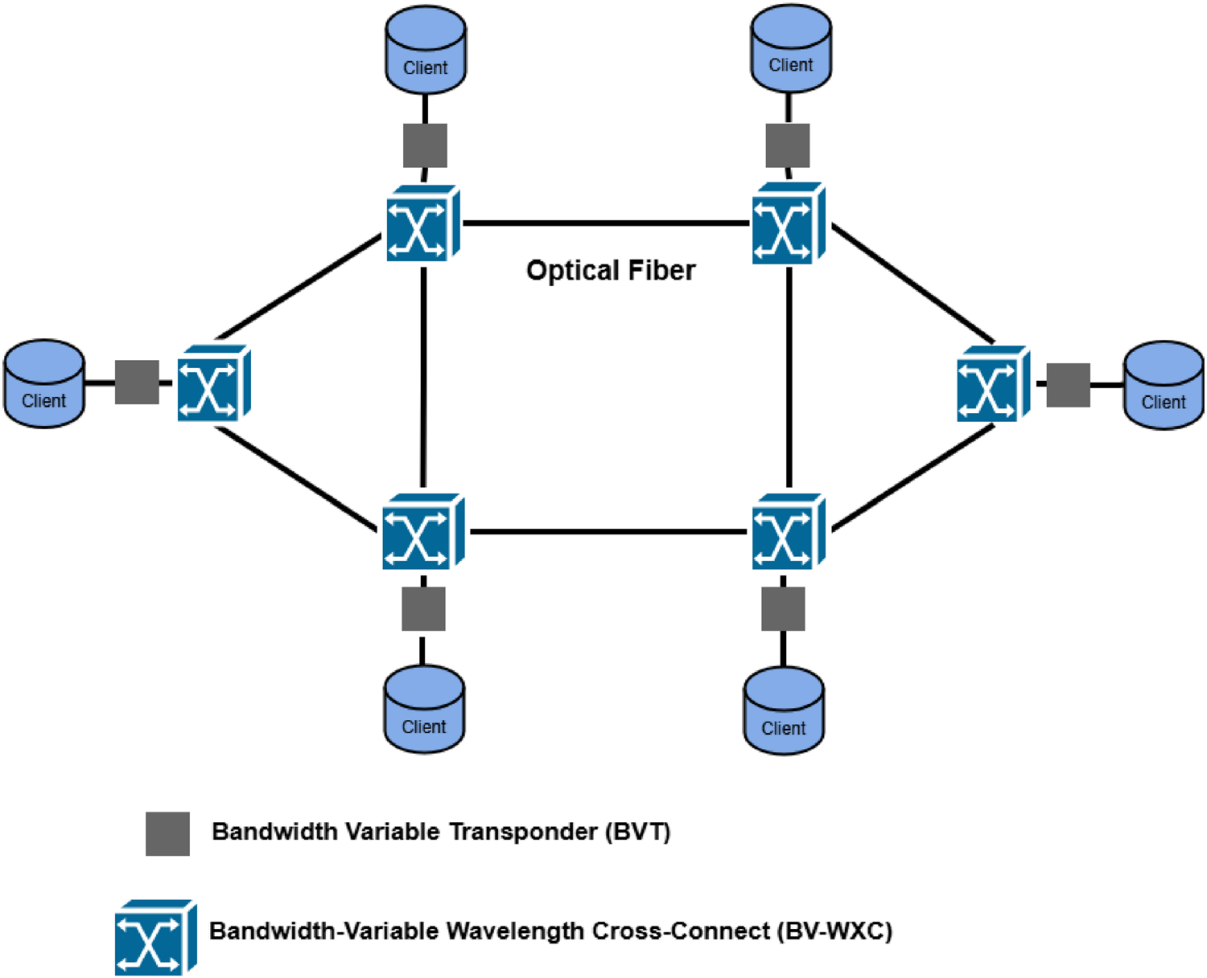
Architecture of an elastic optical network.

In general, the network cost is determined by three network elements viz., node equipment’s (OXC, SSS, splitter/combiner), optical amplifiers, and transponders as shown in Fig. 5. In this subsection, we examine the impact of finer spectrum granularity on the cost and complexity of network elements, which significantly influence both network deployment and operational cost^32^.

The cost and complexity of node equipment, such as OXCs based on WSS or SSS technology, primarily depend on the switch scale. In MEMS-based OXCs, the hardware requirements are closely tied to the number of MEMS mirrors, which directly determines the switch scale and thus significantly impacts the overall cost and complexity of the OXC. The number of MEMS mirrors required by an elastic OXC is determined using Eq. (11)^23^.

11 $$\begin{aligned} N_M = x\times S_{f}\left( 1+ \left[ \frac{x-1}{M_{s}} \right] \right) \end{aligned}$$

where $N_{M}$ denotes the total number of MEMS mirrors, *x* denotes the number of input/output fiber ($x>{0}$), $M_{s}$ denotes the maximum size of the selective switch (i.e. port count). For analysis, the port count is assumed to be $M_{s}$ = 22 and *x* = 1. $S_{f}$ represents the number of FSs per fiber and $C_{f}$ is the total transmission bandwidth of the fiber.

The number of FS ($S_{f}$) can be expressed in terms of slot width ($f_{slot}$) using Eq. (12).

12 $$\begin{aligned} S_{f}=\frac{C_{f}}{f_{slot}} \end{aligned}$$

Substituting Eq. (12) into Eq. (11), we obtain:

13 $$\begin{aligned} N_M = x\times \frac{C_{f}}{f_{slot}}\left( 1+ \left[ \frac{x-1}{M_{s}} \right] \right) \end{aligned}$$

**Fig. 6:**
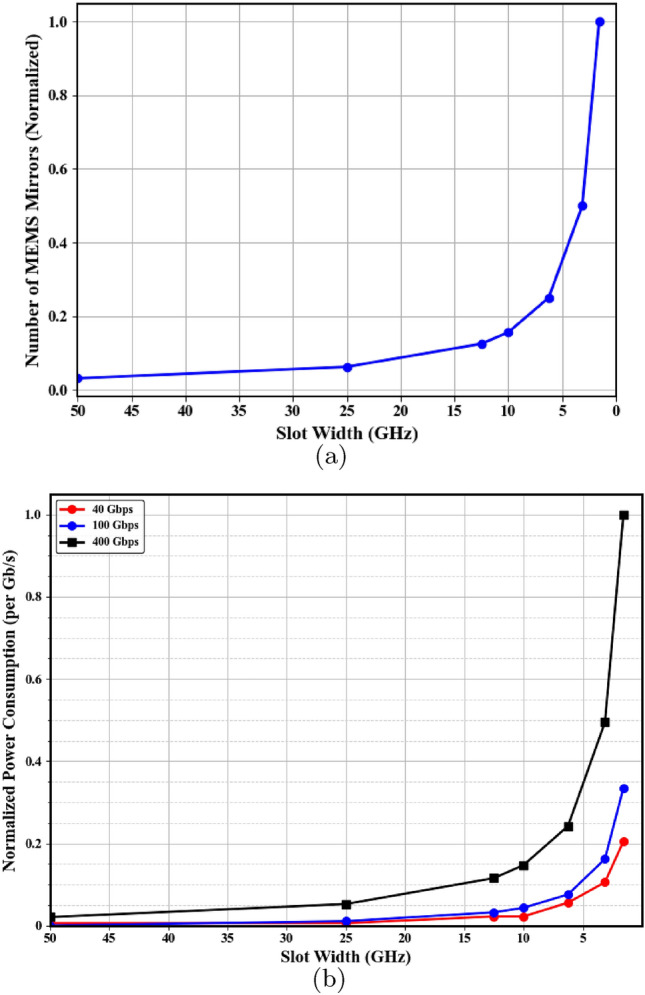
(**a**) Number of MEMS mirrors (Normalized), (**b**) Normalized power consumption with respect to variation in slot width.

This equation reveals a clear inverse relationship between the slot width $f_{slot}$ and the MEMS mirror count $N_{M}$. Reducing the FS width ($f_{slot}$) increases the number of available FSs per fiber, thereby enabling finer spectral granularity. However, this improvement comes at a cost. A smaller $f_{slot}$ requires a greater number of MEMS mirrors, which serve as the switching elements within the OXC. As the number of MEMS mirrors increases, the complexity, cost, and power consumption of the OXC rise significantly. Therefore, while reducing the FS width enhances flexibility and spectral efficiency, it also introduces substantial design and operational challenges. Figure 6aillustrates the normalized value of several MEMS mirrors as a function of slot width. It also signifies the switch scale requirement of the OXCs.

Next, the Bandwidth Variable Transponder (BVT) is a key component of the EON. The flexibility of EONs is achieved through the BVT’s ability to support variable attributes such as modulation techniques, dynamically adaptable coding, data rates, and flexible spectrum occupancy. The power consumption by BVT is evaluated using Eq. (14).

14 $$\begin{aligned} P_{t}= v\times \left( P_{static} +b\times P_{dynamic} \right) \times P_{management} \end{aligned}$$

where $P_{t}$ is the total power consumed by the BVT, *v* represents the number of active lasers, *b* is the operating baud rate of the transponder (in Gbaud), $P_{static}$ denotes the static power consumption (in Watts), $P_{dynamic}$ denotes the dynamic power consumption per unit baud rate (in W/Gbaud), $P_{management}$ denotes the management overhead factor (assumed to be 20% additional power). The values of $P_{static}$ and $P_{dynamic}$ are fixed at 145 W and 5.2 W/Gbaud, respectively.

Assumption: In the adopted BVT architecture, each subcarrier requires a dedicated laser source. Thus, the number of active lasers *v* is equal to the number of subcarriers $N_{sub}$ assigned to a lightpath.The estimation of $N_{sub}$ is given by the Eq. (15).

15 $$\begin{aligned} N_{sub} = \frac{C_{t}}{b_{mk} \times f_{slot}} \end{aligned}$$

where $C_{t}$ represents the size of traffic volume and $b_{mk}$ represents the bit rate per symbol for the selected modulation format. Based on the Eq. (15), the proportional relationship is established, as presented in Eq. (16)^33^:

16 $$\begin{aligned} v\propto \frac{1}{f_{slot}} \end{aligned}$$

This expression demonstrates that the number of active lasers *v*, and consequently the total power consumption of the BVT, is inversely proportional to the FS width $f_{slot}$. As the slot width decreases to achieve finer spectral granularity, the number of required sub-carriers increases, which in turn necessitates more active laser sources. This results in a substantial increase in both the BVT’s power consumption and its associated operational costs. Fig. 6b illustrates the normalized power consumption of the transponder as a function of slot width with the line rates of 40/100/400 Gb/s^34^.

Furthermore, the optical power requirement of an optical fiber depends on the channel count in the fiber. As the transition from a fixed grid to the flexible grid or finer spectrum granularity causes an increment in the number of channels per fiber. As the channel count rises, the total optical power injected into the fiber can approach or exceed the power handling capacity of optical amplifiers. If the amplifiers are not upgraded to handle this increased load, the system may experience signal degradation due to amplifier saturation, nonlinear effects, or power limitations. Therefore, achieving finer spectral granularity often requires higher-power optical amplifiers or more sophisticated power management, which can significantly increase network costs. Moreover, it also increases the software tribulations in the controlling and management system of the network, further leading to increment in the cost and complexity^35^

### Estimation of network cost

In this section, we have computed the network cost based on network parameters. The selection of network parameters has a huge effect on network performance. The optimal use of resources is essential for getting optimal performance. The analysis of network performance and its cost not only gives the network provider an idea to meet demands but also helps in resource allocation while scaling the network. We have modeled the network cost function as a Mixed Integer Linear Programming (MILP) problem to get the minimum network cost based on network parameters and resource utilization. The offline computation gives us the leverage to see the variation in-network cost for different granularities. There are two main contributors to the network cost: (a) hardware requirement (HR) and (b) spectrum consumption (denoted as $\mu _s$).

Both HR and $\mu _s$ are directly influenced by the network load, while $\mu _s$ is also a function of the slot width ($f_{slot}$). since larger slot widths lead to more spectrum consumption. This occurs because (as mentioned in the earlier section) larger slot width causes wastage of spectrum for smaller data rates but with the least hardware complexity. Conversely, with the smaller slot width, the bandwidth allocation can be precise, but the hardware complexity increases to a huge extent. The HR is indirectly affected by the slot width, as variations in slot width alter the accepted traffic, which in turn impacts resource utilization. Thus, to understand this trade-off, we have formulated a cost function that considers the hardware complexity and corresponding network consumption. The objective is to minimize the overall network cost given in Eq. (17). The constraints are given in Eqs. (18), (19), (20) and (21).

Objective: Minimize

17 $$\begin{aligned} \frac{1}{|V |} \sum _{n=1}^{|V |} \left( \frac{T_n}{T_{max}} + \frac{E_n}{E_{max}} \right) + \mu _s \end{aligned}$$

### Parameters and variables

- $T_n$: Number of transponders used at node *n*
- $T_{max}$: Maximum number of transponders at a node
- $E_n$: Number of node equipment used at node *n*
- $E_{max}$: Maximum number of node equipment units at a node
- $\mu _s$: Network spectrum utilization factor
- $\mu _{se}$: Spectrum utilization on link *e*
- $F_e$: Maximum frequency slot index used on link *e*
- $$f_{slot}$$: Slot width
- *B*: Total available bandwidth per link
- |*V*|: Total number of nodes in the network
- *C*: Total number of connections
- $Q_c$: Data rate of the *c*-th connection
- $$B_T$$: Transponder bandwidth
- $$N_g$$: Guard band

Constraints:

18 $$\begin{aligned} T_{n}= \sum _{c=0}^{C} \Bigg \lceil \Bigg \lceil \frac{Q_{c}}{f_{slot}\times M } + N_{g}\Bigg \rceil \times \frac{f_{slot}}{B_{T}} \Bigg \rceil \end{aligned}$$

19 $$\begin{aligned} \mu _{se} = \frac{F_e \times f_{slot}}{B}, \quad \forall e \in E \end{aligned}$$

20 $$\begin{aligned} \mu _s = \max _{e \in E} \left( \frac{F_e \times f_{slot}}{B} \right) \end{aligned}$$

21 $$\begin{aligned} 0 \le F_e \le \left\lfloor \frac{B}{f_{slot}} \right\rfloor \end{aligned}$$

The cost function varies when the fiber capacity is changed. The computation based on fiber bandwidth is avoided to keep the cost calculation easy for understanding. The signals are transmitted by imposing strict constraints to maintain proper Quality of Transmission (QoT). The regenerators are used when the length of the path exceeds the transmission reach of the lowest modulation level, i.e. PM-BPSK. If the re-generator is not utilized, then the call needs to be blocked.

The HR includes all the network elements that are the functions of the slot width. As discussed in subsection “Network cost”, we have calculated the actual number of hardware required for the proper transmission. The average is taken over a number of nodes ($|V |$) to keep the cost computation averaged per node. It is useful while scaling the model for different values of *V*. We have considered the ratio of the actual number and the maximum number of hardware, such that all the network parameters remain on the same level for calculation.

For, e.g. based on the traffic and slot width the number of transponders in use for $n^{th}$ node vary from 0 (not used $(T_n = 0)$) to $T_{max}$ (full use of transponders $(T_n = T_{max})$). Similarly, $E_n$ represents the required node equipment at node *n*, contributing to the overall hardware requirement in the network. The total number of transponders per node is estimated using Eq. (18). The $E_n$ value is calculated based on the explanation in “Network cost”.

The spectrum consumption factor $\mu _{s}$ depends on the slot width and is indirectly influenced by the network traffic through spectrum allocation. It is the ratio of the maximum value of the FS index used on all of the links and the total number of FSs. In other words, $\mu _s$ is the maximum value of $\mu _{se}$, where $\mu _{se}$ represents spectrum utilization on link *e*. Equations (19) and (20) define $\mu _{se}$ and $\mu _s$, respectively.

The contribution of $\mu _s$ in the cost function captures the effect of slot width variation. In addition, due to the direct relationship between $\mu _s$ and $f_{slot}$, $\mu _s$ affects the cost the most. Also, when the slot width is increased (or decreased), the spectrum consumption does not increase (or decrease) linearly. The variables $T_n$ and $E_n$ are bounded by their respective maximum values, while the spectrum utilization factors $\mu _s$ and $\mu _{se}$ lie between 0 and 1.

The cost function considers the number of nodes to calculate the average cost of the network per node. Thus, this cost function is scalable to any network size. The static cost calculation helps to get the least value of the cost for the dynamic network scenario. The MILP model operates on a predefined set of connection requests, which are sorted in descending order of data rate, labeled as $Q_c$ and then sequentially assigned to the network.

This formulation is based on real-world observations that the network cost grows with increased hardware deployment and spectrum fragmentation. As the slot width decreases, spectral efficiency improves (lower $\mu _s$) but requires finer hardware (higher $T_n$, $E_n$). Conversely, larger slots lower hardware requirements but waste spectrum. Our MILP explicitly encodes this tension, allowing the discovery of a sweet spot (as shown in the results section, where 6.25 GHz offers the best trade-off). We model the network cost function using a MILP formulation to capture the interplay between hardware utilization and spectrum usage, both of which are vital to optimizing EONs. The formulation enables us to quantify the trade-offs involved and identify the optimal configuration for minimal cost.

To further clarify the MILP formulation presented in Eqs. (17)–(21), we emphasize that each term in the objective function is carefully chosen to capture the key contributors to network cost in flexible-grid EONs. The normalized transponder usage $\frac{T_{n}}{T_{\text {max}}}$ and node equipment usage $\frac{E_{n}}{E_{\text {max}}}$ collectively reflect the hardware footprint per node, which significantly influences capital and operational expenditure. The spectrum consumption term $\mu _s$ captures the efficiency of FS utilization across the network and represents the impact of slot width on spectrum fragmentation. Together, these terms model the trade-off between spectrum efficiency and hardware complexity. The constraints ensure feasible and implementable solutions by accounting for hardware capacity, spectrum continuity, and slot indexing limits. This formulation allows us to quantify and compare network cost under different granularity scenarios, enabling the identification of an optimal slot width that balances blocking performance with cost-effectiveness.

## Performance analysis

In our simulation platform, the slot width is adjusted from 50 GHz down to 1.5625 GHz. For the C-band, each fiber link has a total bandwidth of 4 THz, which accommodates 80, 160, 320, 400, 640, 1280, 2560 FSs, respectively. When considering the combined C + L band, the total bandwidth extends to 8 THz, which accommodates 160, 320, 640, 800, 1280, 2560, and 5120 FSs, respectively, for the same slot widths. We have generated $10^{5}$ bidirectional connection requests that arrive following a Poisson process with an average arrival rate of $\lambda$ requests per unit time, and their holding times follow a negative exponential distribution with a mean of $\frac{1}{\mu }$ time units. Hence, the traffic load is estimated as $\frac{\lambda }{\mu }$ Erlang. For each connection request, source (*s*) and destination (*d*) nodes are randomly selected from all possible node pairs in the network. Spectrum assignment is performed using the First-Fit (FF) policy, which is chosen for its simplicity, low computational overhead, and widespread adoption as a baseline in EON studies, ensuring consistent and fair performance evaluation. A single FS ($N_{g}$ = 1) is reserved as the guard band for each connection.

In our simulations, the blocking probability analysis is performed using both low-data-rate and high-data-rate connection demands across all network scenarios. The traffic loads are set as follows: 300, 400, 500, and 600 Erlangs for NSFNET, 400, 500, 600, 700 Erlangs for the COST 239 topology and 800, 1000, 1200, and 1400 Erlangs for the USNET topology. The evaluation is conducted for both single-band (C-band) and multi-band (C + L-band) configurations. The traffic load is varied in discrete steps across a moderate-to-high range, following established practices in EON performance evaluation^36,37^. Low-data-rate connections include 12.5 Gb/s, 25 Gb/s, 40 Gb/s, 100 Gb/s, while the high–data-rate connections include 200 Gb/s, 400 Gb/s, 800 Gb/s, 1000 Gb/s.

For the BP-Cost (Blocking probability and Cost) tradeoff analysis, simulations are conducted on the NSFNET and the Pan-European networks. In this case, connection data rates are set to 12.5 Gb/s–200 Gb/s, and the offered traffic load varies from 500 to 800 Erlangs.

This separation of scenarios allows blocking probability to be evaluated on three realistic backbone networks (NSFNET, USNET, and the COST 239 topology) for both low and high data rates in two spectral domains (C band and C + L band), while BP-cost tradeoffs are studied on the NSFNET and Pan-European reference networks. In the cost analysis, although the absolute cost depends on the specific topology, the qualitative trend—total network cost increasing with decreasing slot widths—has been observed to be similar across the realistic backbone topologies considered.

All simulations were implemented using C++, MATLAB, and Python on a personal computer equipped with an Intel Xeon 2.67 GHz CPU and 16 GB of RAM. The MILP was implemented using IBM CPLEX and executed in Visual Studio 14.0 (2015). To ensure statistical reliability, each traffic load was tested over 10 independent runs, and the results were reported with a 95% confidence interval and an error margin within 5%. Table 1 provides a summary of the simulation parameters. The transmission reach values for different modulation formats are adopted from^38^.

**Table 1:** Simulation parameter.

Parameter	Value
Bandwidth of a frequency slot, $(f_{slot})$ (GHz)	50 GHz down to 1.5625 GHz
Spectral band	C-band/C+L band
Link capacity (THz)	4 THz (C-band)/8 THz (C + L band)
Transmission reach of BPSK (km.)	5000
Transmission reach of QPSK (km.)	2500
Transmission reach of 8-QAM (km.)	1250
Transmission reach of 16-QAM (km.)	625

**Fig. 7:**
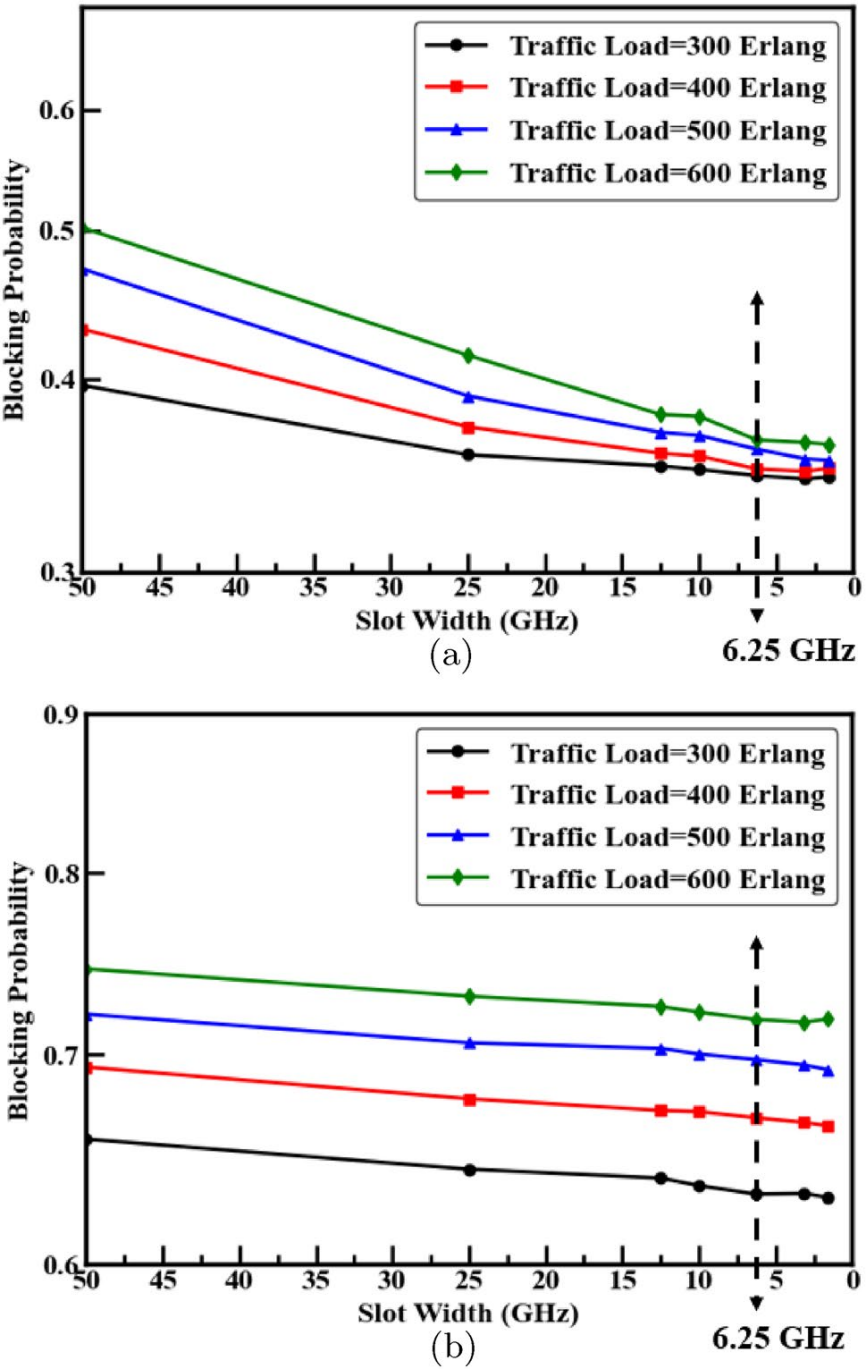
Blocking probability vs. Slot width for NSFNET in the C-band for two data-rate scenarios: (**a**) low data rate (12.5 Gb/s, 25 Gb/s, 40 Gb/s, 100 Gb/s) and (**b**) high data rate (200 Gb/s, 400 Gb/s, 800 Gb/s, 1000 Gb/s).

### Simulation results and discussion

#### Blocking probability results

This section presents the blocking probability results for the three representative topologies: NSFNET, USNET, and COST 239. The evaluation covers both low and high data rates and includes performance in the C-band and the C + L band. Together, these results provide a comprehensive assessment of how slot-width granularity impacts blocking behavior across different network topologies and spectral regions.

Figure 7a,b illustrate blocking probability variation with slot width in the C-band for NSFNET topology under different traffic loads for low and high data rates, respectively.

Here, we determine the blocking probability by varying the slot width from 50 GHz down to 25 GHz, 12.5 GHz, 10 GHz, 6.25 GHz, 3.125 GHz, and 1.5625 GHz at different traffic loads. Reducing the slot width or getting finer spectrum granularity leads to a reduction in blocking probability. But another interesting observation is that on further reducing the slot width from 6.25 GHz to 3.125 GHz and later to 1.5625 GHz, it yields only marginal improvement, and at very narrow slot widths, fragmentation may cause a slight increase in blocking, particularly at high data rates. This counterintuitive behavior is due to the fact that narrower slots require a larger number of contiguous slots to support the same high data rate. In a dynamically loaded network, such fine-grained slot allocation can result in higher spectral fragmentation, making it increasingly difficult to find large, continuous spectrum blocks. Consequently, despite offering finer granularity, smaller slot widths can degrade performance by increasing the likelihood of blocking, particularly for high data rate connections in fragmented spectrum conditions. These results form a baseline trend against which the other topologies are compared.

Next, we present the results for the USNET topology, again under both low and high data-rate scenarios. USNET is larger and more densely connected than NSFNET, and including both traffic levels enables us to verify that the trends identified in NSFNET also hold in a more complex network.

**Fig. 8:**
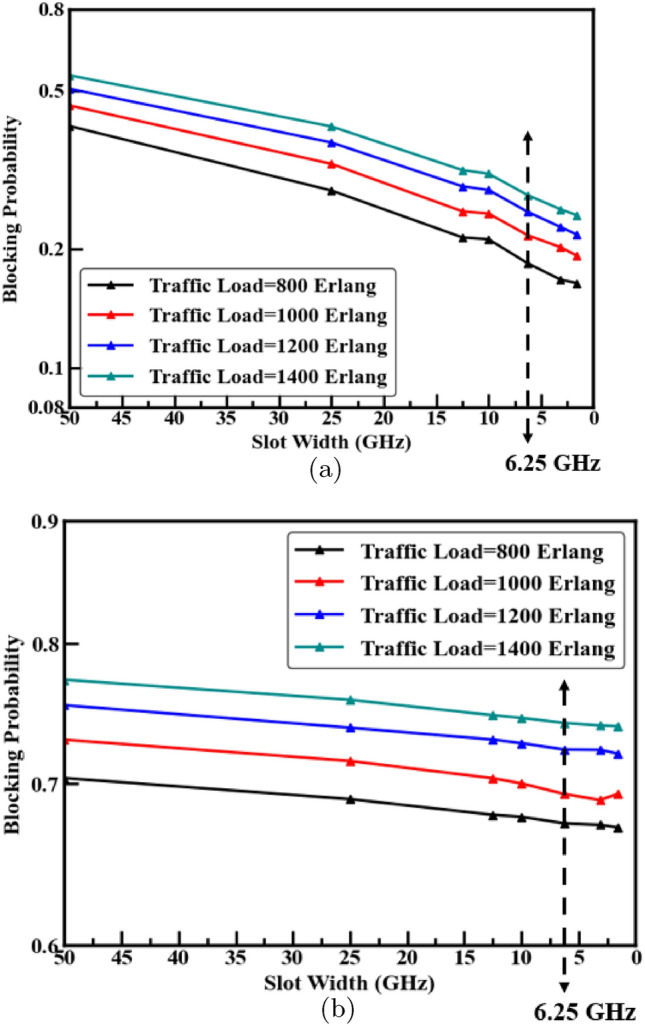
Blocking probability vs. Slot width for USNET in the C-band for two data-rate scenarios: (**a**) low data rate (12.5 Gb/s, 25 Gb/s, 40 Gb/s, 100 Gb/s) and (**b**) high data rate (200 Gb/s, 400 Gb/s, 800 Gb/s, 1000 Gb/s).

The USNET results, shown in Fig. 8a,b for low and high data rates, respectively, exhibit a similar pattern to NSFNET while reflecting the characteristics of a larger and more highly connected network. The reduction in slot width improves blocking performance up to 6.25 GHz, beyond which the gains diminish. The presence of fragmentation at extremely fine granularities is again observed, especially under high traffic loads. Presenting both data-rate levels for USNET confirms that the slot-width trend remains consistent even as network size increases.

**Fig. 9:**
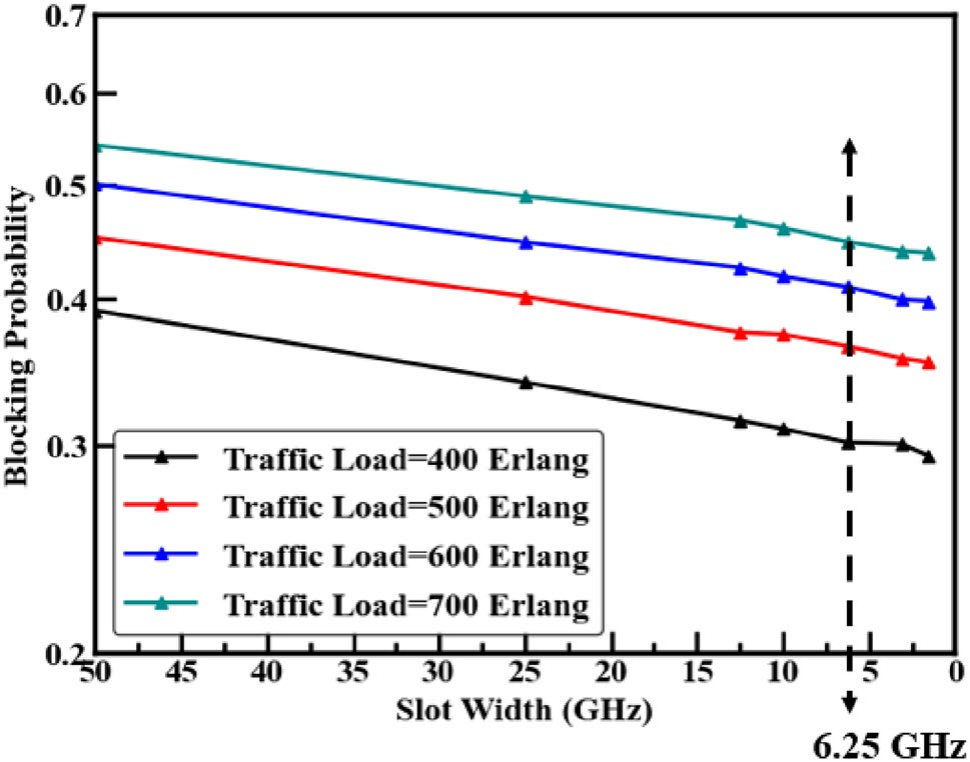
Blocking probability vs. slot width for COST 239 in the C-band for high data-rate scenario (200 Gb/s, 400 Gb/s, 800 Gb/s, 1000 Gb/s).

Next, for the COST 239 topology, the blocking probability results are shown only for high data rates, as indicated in Fig. 9. At low data rates, the blocking probability is negligible across all slot widths, offering no additional insight. The high-rate results reveal the same behavior observed in NSFNET and USNET: the most substantial improvement occurs when reducing the slot width to 6.25 GHz, while finer granularity offers limited performance gain and may introduce fragmentation effects.

**Fig. 10:**
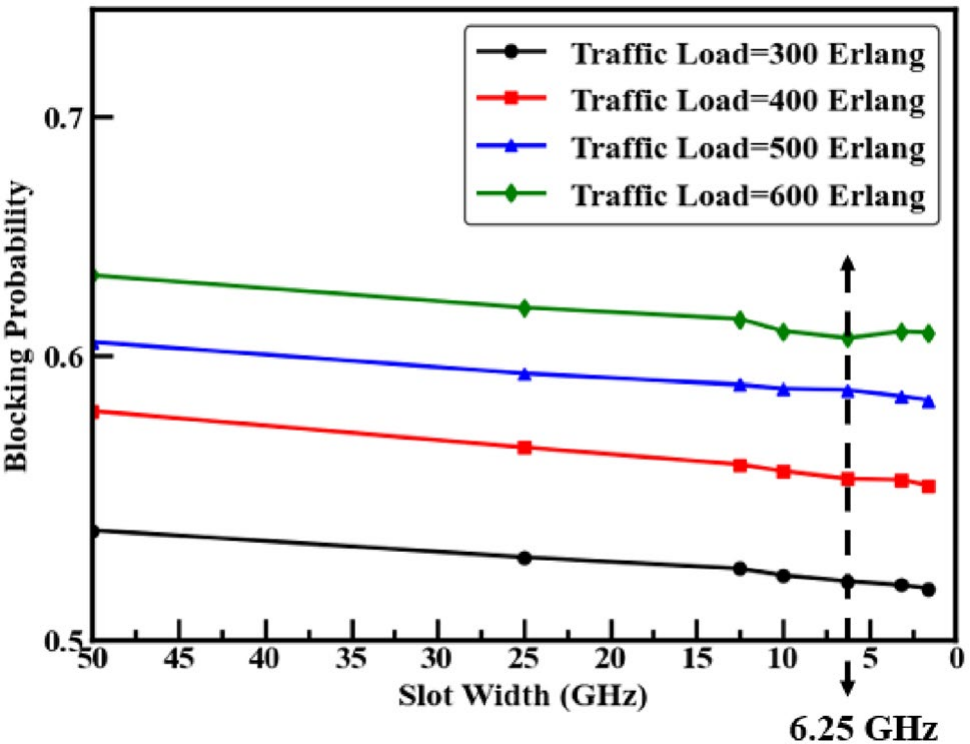
Blocking probability vs. Slot width for NSFNET in the C + L-band for high data-rate scenario (200, 400, 800, 1000 Gb/s).

**Fig. 11:**
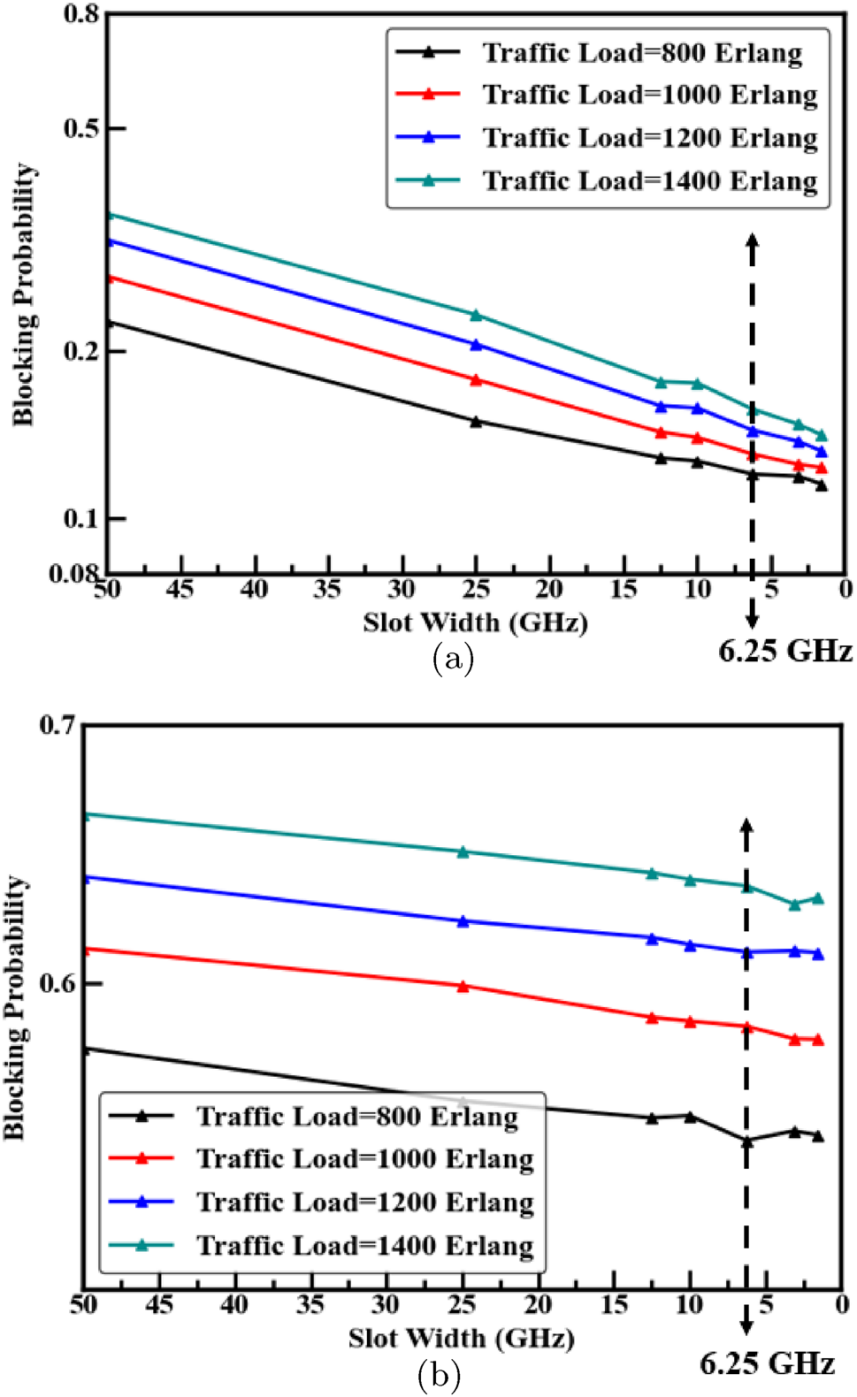
Blocking probability vs. Slot width for USNET in the C + L-band for two data-rate scenarios: (**a**) low data rate (12.5 Gb/s, 25 Gb/s, 40 Gb/s, 100 Gb/s) and (**b**) high data rate (200 Gb/s, 400 Gb/s, 800 Gb/s, 1000 Gb/s).

**Fig. 12:**
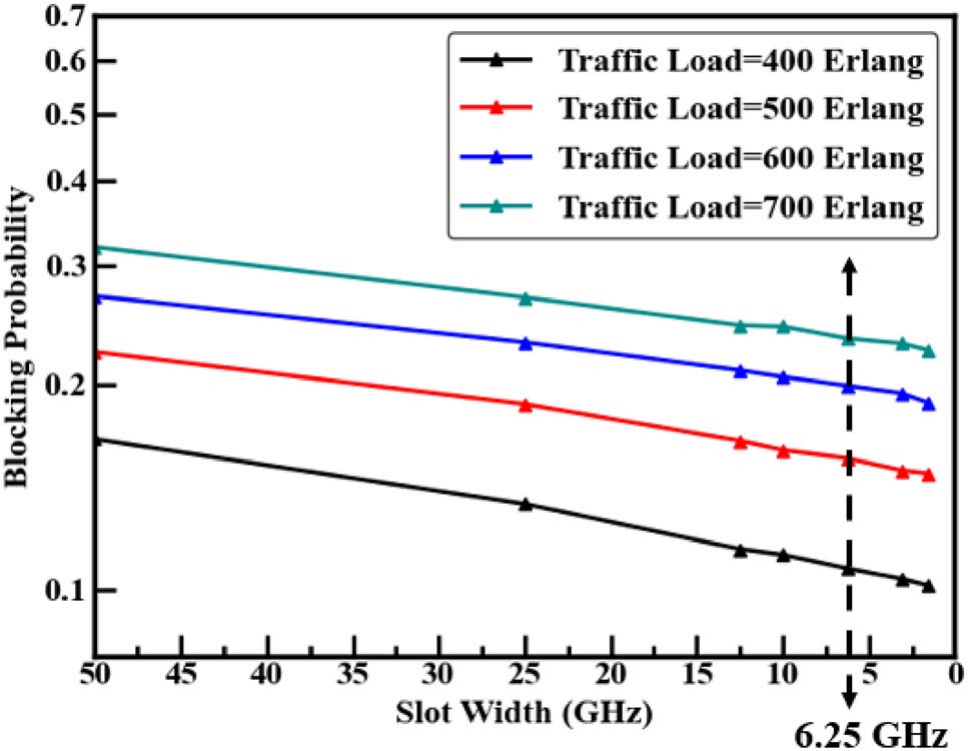
Blocking probability vs. Slot width for COST 239 in the C + L-band for high data-rate scenario (200 Gb/s, 400 Gb/s, 800 Gb/s, 1000 Gb/s).

Similarly, the blocking probability in the C + L band is shown in Figs. 10, 11, and 12 for the NSFNET, USNET, and COST 239 topologies, respectively. Overall, the results confirm that extending the spectrum does not alter the fundamental slot-width trends observed in the C-band.

#### Optimal slot width analysis

In this subsection, we analyze the optimal slot width, considering the network cost and blocking probability.

**Fig. 13:**
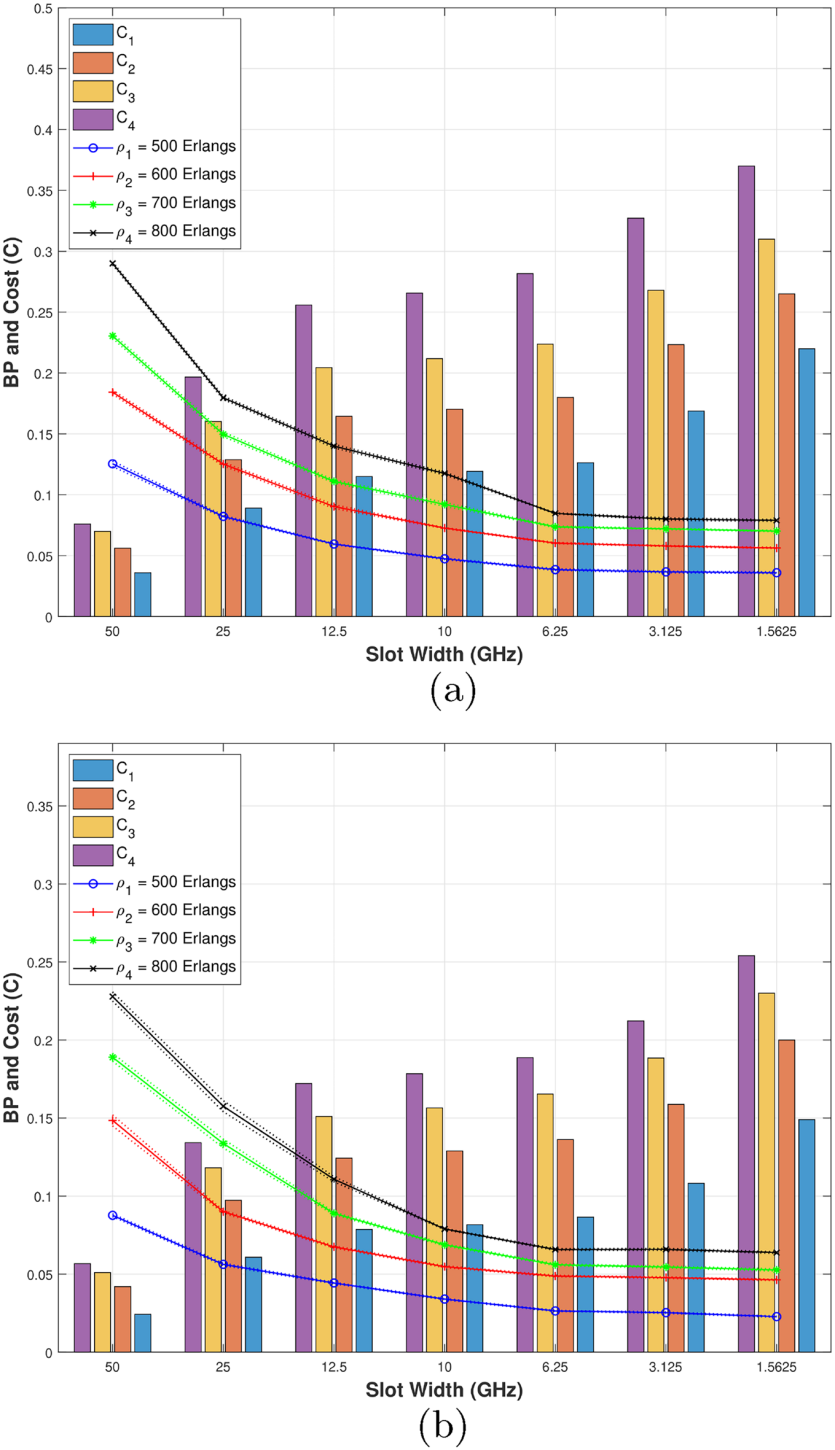
Blocking performance and network cost-based analysis of slot widths in (**a**) NSFNET network, and (**b**) PAN-European network (12.5–200 Gb/s data rates).

Figure 13a,b illustrate the blocking probability and network cost-based analysis of slot width at different traffic loads with the NSFNET and PAN-European topology, respectively. $\rho _{1}$ and $C_{1}$, $\rho _{2}$ and $C_{2}$, $\rho _{3}$ and $C_{3}$, $\rho _{4}$ and $C_{4}$ represent the blocking probability and network cost at traffic loads 500 Erlangs, 600 Erlangs, 700 Erlangs, 800 Erlangs respectively. Here, a decrement in the slot width leads to a reduction in the blocking probability and an increment in the network cost. Up to a slot width of 6.25 GHz, spectrum savings help mitigate the impact of additional increases in network cost. However, further reducing the slot width from 6.25 GHz to 3.125 GHz, and then to 1.5625 GHz, results in negligible improvements in blocking probability or spectrum savings, while the network cost increases significantly.

**Fig. 14:**
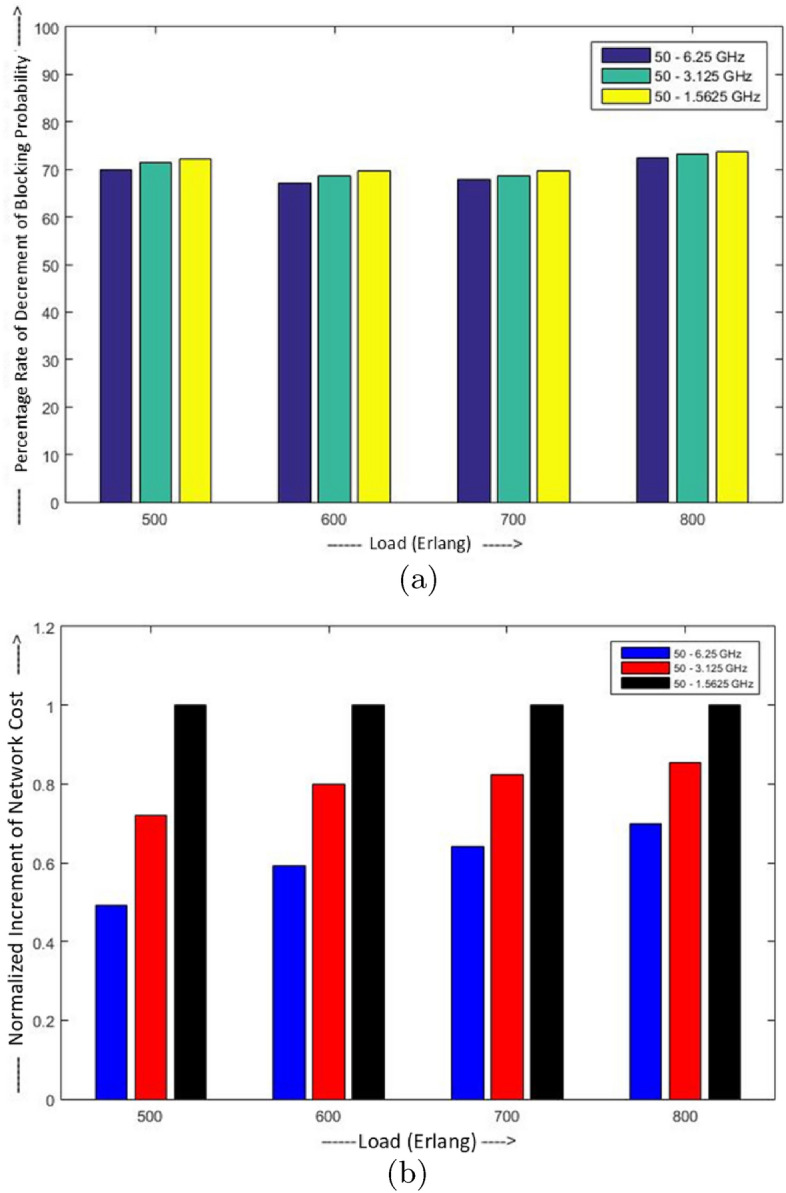
NSFNET topology: (**a**) Percentage rate of decrement of blocking probability, and (**b**) Normalized increment in network cost by varying the slot width from 50 GHz to 6.25 GHz, 50 GHz to 3.125 GHz, and 50 GHz to 1.5625 GHz.

**Fig. 15:**
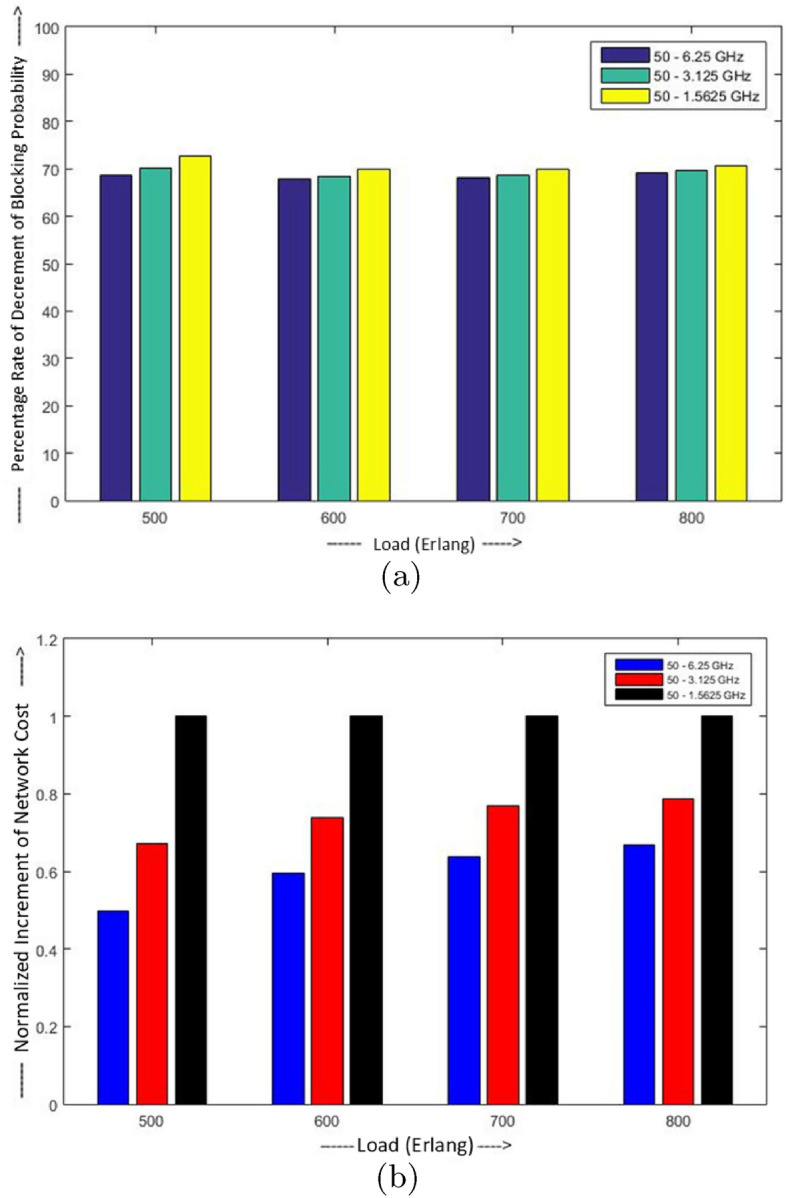
Pan-European network topology: (**a**) Percentage rate of decrement of blocking probability, and (**b**) Normalized increment in network cost by varying the slot width from 50 GHz to 6.25 GHz, 50 GHz to 3.125 GHz, and 50 GHz to 1.5625 GHz.

Figure 14a,b illustrate the rate of reduction in blocking probability and the normalized increment in network cost for the NSFNET topology, respectively. Similarly, Fig. 15atopology, respectively. In this analysis, we evaluate the performance of various slot widths, 6.25 GHz, 3.125 GHz, and 1.5625 GHz, by comparing them against a conventional baseline of 50 GHz. The evaluation focuses on two critical metrics: the percentage improvement in blocking probability and the normalized increment in network cost. The objective is to identify the slot width that offers the best trade-off between performance and efficiency.

Our simulation results demonstrate that reducing the slot width from 50 GHz to 6.25 GHz leads to a significant improvement in blocking probability, indicating more efficient spectrum utilization. However, further reduction to 3.125 GHz and 1.5625 GHz results in diminishing returns. The additional gain in blocking probability becomes marginal or negligible, while the network cost increases substantially due to the higher number of finer-grained slots that must be managed. This increase in cost includes not only the physical resources (e.g., transponders and switching complexity) but also operational overhead related to routing and spectrum management at finer granularity.

These findings are consistent across both the NSFNET and Pan-European topologies, reinforcing the conclusion that the optimal balance point is achieved at 6.25 GHz. This slot width provides a favorable compromise by delivering substantial performance benefits without incurring disproportionate increases in network cost or complexity. As such, 6.25 GHz emerges as a practical and cost-effective configuration for elastic optical networks under the traffic conditions studied.

## Conclusion

In this paper, we identified the optimal slot width for efficient utilization of spectral resources while maintaining low hardware and software complexity. The analysis shows that a slot width of 6.25 GHz offers the best balance between blocking probability and network cost, resulting in a notable performance improvement with a reasonable cost increase. From a practical viewpoint, these results give useful advice for network designers and operators by measuring the trade-offs between slot width, blocking performance, and cost in elastic optical networks. This can help make better decisions in network planning and upgrades.

Future work will aim to develop more detailed cost models that include more network components and operational factors. Extending the analysis to different network topologies will further confirm the broad applicability of the approach. Also, integrating new technologies like Software-Defined Networking (SDN) and machine learning could lead to more flexible and efficient spectrum management for real-time network environments.

## Data Availability

The data that support the findings of this study are available from the corresponding author upon reasonable request.

## Code availability

The simulation code used to generate the results of this study is available from the corresponding author upon reasonable request.
